# Discussion

**Published:** 1918-10

**Authors:** 


**Discussion.**

Colonel P. B. Soltau, R. A. M. C., said that the high rate of respiratory infections in the American army had been pointed out. As divisions go more and more in line they may be exposed to gas. The British experience has been that when men were suffering from broncho-catarrh the death-rate from gas infection tended to increase.

If the high rate of respiratory infections in American troops is maintained, it might cause a greater number of deaths from mustard gas in troops exposed to that agent. This acts as a chemical irritant and sets up a burn which is followed by bacteriological infection, especially in the pleura. The bronchi will get burned from the vapor and broncho-pneumonia, which will kill a great number of men, is a likely consequence.

There should, therefore, be a special effort to improve the health of the men by avoiding crowding.

Colonel Cummins, R. A. M. C., said that he desired to lay particular stress on one disease that had been mentioned, and that was tuberculosis. He did not wish to lay stress on it so much in reference to white troops as to black troops.

America has a complete army of black troops, and one could not help being struck with a point of this kind. Professor Borel had brought out clearly the fact that the blacks come to this country with no real protection against pneumococcus infections and that they have no resistance. Major Zinsser said how, under hygienic conditions, resistance could be increased and how it could be lost through defective sanitation. These remarks, made in reference to pneumonia, should be applied to tuberculosis.

It has been quite clearly shown that tuberculosis is a very fateful disease to the blacks as a race, when they are living under native conditions. The speaker thought the important point to be this: It is clearly the duty of our officers to improve sanitation for the colored troops and to protect them from tuberculosis while they are fighting or working in the war, so that they may not go home and spread this disease among their own people.

Overcrowding is the principal factor in this disease and it should be decreased. We should serve the black race and should exercise our right to preserve them from a disease that we know how to fight.

Médecin-Major Borel said that he wished to supplement what the previous speaker had said on the subject of tuberculosis. He wanted first to present statistics on the vaccination of a battalion against pneumonia. The battalion comprised 7000 men of from two to three years enlistment. Tuberculosis broke out in it. At this time some fresh battalions arrived from the colonies. In general, only one or two soldiers of the new troops were affected by tuberculosis.

The speaker used tuberculin on the soldiers of one of the fresh battalions. No reaction was obtained. On the contrary, in the case of the troops which were returning from the front and which had passed from two to three years in France, the following results were observed: in 10 cases there were three positive reactions; one of the men had just spent two months in the hospital; another had been gassed; and a third had been severely wounded.

Tuberculosis among the colored troops does not come from the colony, and is very rarely found among new arrivals; and if it is found, by chance, the sufferer is from a town where French is spoken.

The speaker said that he now passes all the new arrivals in review with the bodies exposed to the waist. The inspection is rapid but sufficient. The characteristics of tuberculosis among the colored troops are easily distinguished. The skin of a colored man with symptoms of tuberculosis is dry and withered instead of being shiny. If, furthermore, there are any cervical ganglions the individual is withdrawn from the ranks and hospitalized for an observation period of 15 days, and is then sent back to the colony.

The principle followed is to inspect the battalions every month, and to isolate and watch any suspected cases.

There is a special service for hunting out tuberculosis at St. Raphael. Out of the battalion of 7000 men there were 47 cases of tuberculosis or of pneumonia in three months. In other battalions there was not a single case.

Colonel Thayer said that Professor Borel had noted that of the Africans who arrive in France a very small proportion had tuberculosis. There is evidence that some of them acquire it here. Professor Borel has adopted the method of carefully examining the men when they arrive and of inspecting them every month, setting aside all suspected cases.

Before sending American troops over here a careful study was made in America and about 1 o/o of the men were found to show signs of tuberculosis and were kept at home. Since we have been here we have had but few cases. Major Webb has pointed out that the disease is beginning to appear among our troops in France. It is only by carefully examining the men that it can be kept down.

Colonel Beveridge, in closing, said that a certain proportion of our colored troops (Kaffirs and Chinese) come to this country susceptible to tuberculosis. Under conditions of climate and exposure, the disease develops. In the French army it has been noticed that certain men get tuberculosis after exposure to gas.

Mumps and measles follow the seasons. Last year we had less cases than in 1916, and this year less again. One generally has one attack of mumps and measles during life; therefore, most men are immune because they have had these diseases in childhood. The cases we have had were chiefly in men coming from country districts, town men having had these infections in childhood. It has also been noted that men from the mountain districts of Scotland get mumps or measles when they mingle with other men.

Outbreaks of scarlet fever in our army have been small. Diphtheria has been increasing in the British forces during the last two years and we get a good deal of it in hospitals. The speaker had been told that it occurred chiefly among the nurses and orderlies, and was presumably due to infection brought by the wounded.

Diphtheria frequently break out among children, and men get it in this way. Good results have been obtained by isolating the cases.

A certain amount of infection is carried by the sharing of blankets. It would be ideal if a man could always retain his blanket, for there is always great danger if one man passes on his blanket to another.

MAJOR HAVEN EMERSON, M. R. S, said that reports from the Chief Surgeon show an increase of streptococcus infection in the American forces. There have recently been cases of streptococcus pneumonia, with 15 deaths, occurring from much overcrowding. It is interesting to know that the same conclusions as arrived at during the meeting had been arrived at by division surgeons in various parts. They have used various devices to diminish the over-crowding and efforts have been made to get the men out of crowded billets and into tents. In sanitary reports it has been mentioned that prevention of pneumonia is in proportion to the ingenuity of the sanitary inspectors in obtaining more space than that allotted.

In hospitals principally the Schick test should be made : this has saved certain organizations from infection. Recently there have been 10 cases of diphtheria among the attendants in one hospital.

In regard to the question of the country man and the city man, there is some evidence of a good deal of duplicate infection. Some men, who believed they had had measles in childhood and who were exposed to heavy infection, have had secondary attacks.

It is worth noting that prompt action resulting from rapid reporting of cases ensures very little effect locally, and it is necessary to get rapidly into contact with the French medical officer for the civilian population.

GENERAL BURTCHAELL said that one point of importance was the relative amount of cubic space and floor space. Under war conditions it is quite impossible in the majority of circumstances to give the amount of floor space and cubic space laid down in text books; but we must bear in mind that the essential point is to ensure adequate ventilation. Space in itself is not nearly so important as free ventilation.

In the case of dug-outs, ventilation is fairly good, but space is limited and the men get on very well without developing any great amount of infectious diseases.

One cannot help feeling that the part played by blankets in conveying infection is relatively small in the British army because of the few cases of infectious diseases which have developed. The proximity of men in billets and in dug-outs with a small cubic space is a serious matter, but the diseases communicated or developed from over-crowdings generally occur among men living day after day in limited spaces. Soldiers are, as a rule, in a confined space for one night only, and the medical officer is often too insistent that troops cannot occupy shelter because they would be over-crowded. He should consider that when they are to occupy this limited space for one or two nights only it is better to take a limited risk and give them shelter than to expose them to the elements outside.

